# T cell adhesion and cytolysis of pancreatic cancer cells: a role for E-cadherin in immunotherapy?

**DOI:** 10.1038/sj.bjc.6600597

**Published:** 2002-10-21

**Authors:** J J French, J Cresswell, W K Wong, K Seymour, R M Charnley, J A Kirby

**Affiliations:** Applied Immunobiology Group, Department of Surgery, The Medical School, University of Newcastle, NE2 4HH, UK; Department of Surgery, Freeman Hospital, Newcastle upon Tyne, UK

**Keywords:** pancreatic cancer, immunotherapy, cytotoxic, T cells, E-cadherin

## Abstract

Pancreatic cancer is an aggressive and potent disease, which is largely resistant to conventional forms of treatment. However, the discovery of antigens associated with pancreatic cancer cells has recently suggested the possibility that immunotherapy might become a specific and effective therapeutic option. T cells within many epithelia, including those of the pancreas, are known to express the αEβ7-integrin adhesion molecule, CD103. The only characterised ligand for CD103 is E-cadherin, an epithelial adhesion molecule which exhibits reduced expression in pancreatic cancer. In our study, CD103 was found to be expressed only by activated T cells following exposure to tumour necrosis factor beta 1, a factor produced by many cancer cells. Significantly, the expression of this integrin was restricted mainly to class I major histocompatibility complex-restricted CD8+ T cells. The human pancreatic cancer cell line Panc-1 was transfected with human E-cadherin in order to generate E-cadherin negative (wild type) and positive (transfected) sub-lines. Using a sensitive flow cytometric adhesion assay it was found that the expression of both CD103 (on T cells) and E-cadherin (on cancer cells) was essential for efficient adhesion of activated T cells to pancreatic cancer cells. This adhesion process was inhibited by the addition of antibodies specific for CD103, thereby demonstrating the importance of the CD103→E-cadherin interaction for T-cell adhesion. Using a ^51^Cr-release cytotoxicity assay it was found that CD103 expressing T cells lysed E-cadherin expressing Panc-1 target cells following T cell receptor stimulation; addition of antibodies specific for CD103 significantly reduced this lysis. Furthermore, absence of either CD103 from the T cells or E-cadherin expression from the cancer cells resulted in a significant reduction in cancer cell lysis. Therefore, potentially antigenic pancreatic cancer cells could evade a local anti-cancer immune response *in vivo* as a consequence of their loss of E-cadherin expression; this phenotypic change may also favour metastasis by reducing homotypic adhesion between adjacent cancer cells. We conclude that effective immunotherapy is likely to require upregulation of E-cadherin expression by pancreatic cancer cells or the development of cytotoxic immune cells that are less dependent on this adhesion molecule for efficient effecter function.

*British Journal of Cancer* (2002) **87**, 1034–1041. doi:10.1038/sj.bjc.6600597 www.bjcancer.com

© 2002 Cancer Research UK

## 

A number of tumour-associated antigens have been described in pancreatic carcinoma (Mckenzie and Apostolopoulos, 1999). These include MUC-1 (Ho and Kim, 1994), Pancreatic Associated Protein (PAP) (Motoo *et al*, 1998), Her2/neu (Yamanaka *et al*, 1993), and K-ras (Berndt *et al*, 1998). Furthermore, a recent *in vitro* study by Schnurr *et al* (2001) suggests that these antigens may be important for T cell recognition and lysis of autologous pancreatic cancer cells. Despite the ability of autologous T cells to specifically recognize tumour-associated antigens it is clear that antigenic tumours *in vivo* successfully evade the immune system, demonstrated by their proliferative and metastatic nature. Several hypotheses have been proposed to explain the failure of the immune system to clear the cancer cells. These include secretion of immunosuppressive cytokines (Chouaib *et al*, 1997), induction of apoptosis in the infiltrating T-cell population (Igney *et al*, 2000), down-regulation of target cell membrane molecules such as MHC class I and II (Scupoli *et al*, 1996), and down regulation of adhesion molecules (Pettit *et al*, 2000).

Adhesion between lymphocytes and their targets is thought to be a prerequisite for effective recognition and cytotoxicity. Hence, adhesive interactions between pancreatic cancer cells and cytotoxic T cells may be crucial to an effective anti-cancer immune response. The interaction between lymphocyte function associated antigen-1 (LFA-1) expressed by lymphocytes and intercellular adhesion molecule (ICAM-1) on antigen presenting cells is known to be important for adhesion (Takahashi *et al*, 1993). LFA-1 is a member of the β2 integrin family of molecules and has been shown to transduce ‘outside–in’ signalling that can augment T lymphocyte activation (Dubey *et al*, 1995). It has also been demonstrated that the LFA-1→ICAM-1 interaction can increase T-cell cytotoxicity by causing mobilisation of cytotoxic granules, whilst antibody blockade of this interaction causes a reduction in cytotoxicity (Zhou *et al*, 1998). However, it is not clear whether additional adhesion receptor systems can play an important role in this process.

The αEβ7 heterodimer, often referred to as CD103 (an epitope of the α subunit), is a recently discovered member of the integrin family. It is expressed by over 90% of lymphocytes in the intestinal epithelium and 40% of lymphocytes in gut lamina propria (Cerf-Bensussan *et al*, 1987). Similar expression by lymphocytes has been reported in the lung (Rihs *et al*, 1996), salivary gland (Fujihara *et al*, 1999), bladder (Cresswell *et al*, 2001) and pancreatic epithelium (Ebert *et al*, 1998). As the αEβ7 integrin is found on fewer than 2% of circulating lymphocytes (Cerf-Bensussan *et al*, 1987), it is considered a marker for intraepithelial lymphocytes (IEL).

The only characterised ligand for αEβ7 is the cell adhesion molecule E-cadherin. When E-cadherin negative cells were transfected with E-cadherin it was found that a greater proportion of IEL was able to bind; this increase was inhibited by specific adhesion molecule blocking antibodies (Cepek *et al*, 1994). It has also been demonstrated that the αEβ7 integrin, in common with LFA-1, may have co-stimulatory properties (Sillett *et al*, 1999). For example, IEL-mediated cytolysis is enhanced via an unknown intracellular signalling pathway if the αEβ7 integrin is cross-linked with antibody (Roberts and Kilshaw, 1993). Furthermore, IEL have a greater capacity than peripheral blood lymphocytes (PBL) for spontaneous cytotoxicity of epithelial cells (Taunk *et al*, 1992).

E-cadherin is a trans-membrane protein expressed primarily by epithelial cells, where it stabilises the homotypic adhesion crucial for development and maintenance of functioning epithelium (Gumbiner, 1996). Approximately 90% of human malignancies are of epithelial origin. E-cadherin is known to have tumour suppressor effects (Frixen *et al*, 1991; Christofori and Semb, 1999), and reduced expression during cancer development has been observed in many epithelial cancers including breast adenocarcinoma (Berx *et al*, 1995), ovarian carcinoma (Davies *et al*, 1998) and pancreatic adenocarcinoma (El-Hariry *et al*, 1999). In pancreatic cancer, loss of E-cadherin expression is associated with high grade and advanced stage (Pignatelli *et al*, 1994; Karayiannakis *et al*, 1998), and is a marker of poor prognosis (Kuniyasu *et al*, 1999).

As over 90% of mucosal T cells express an adhesive counter-receptor specific only for E-cadherin, it is likely that this interaction performs a physiologically important role within the epithelial compartment. This has not been defined but potentially provides a mechanism to enhance antigen recognition within the epithelium. Indeed, it has been proposed that IEL may be responsible for specific immunosurveillance within anatomically vulnerable epithelia (Kilshaw and Karecla, 1996), having a potential to eliminate both infected and cancerous epithelial cells. Clearly, interaction between the αEβ7 integrin and E-cadherin does not function in isolation and other molecules are likely to be involved in IEL function, including the LFA-1→ICAM-1 system. Although ICAM-1 is absent from gut (Bloom *et al*, 1995) and normal pancreatic epithelium (Shimoyama *et al*, 1997) it has been demonstrated at varying levels on pancreatic carcinoma cells (Shimoyama *et al*, 1997), and its expression can be increased by TNFα and IFNγ stimulation (Shimoyama *et al*, 1999).

The current study was designed to examine the role of the αEβ7→E-cadherin interaction in pancreatic cancer. An E-cadherin negative pancreatic carcinoma cell line was transfected with human E-cadherin cDNA. Cells expressing E-cadherin were selected for investigation of the potential of this molecule to increase the adhesion of T cells that had been induced to express the αEβ7-integrin. Following definition of the conditions required for T cell adhesion, a series of experiments was performed to determine whether interaction with E-cadherin can increase the lysis of pancreatic cancer cells by CD103+ T cells.

## MATERIALS AND METHODS

### Antibodies

Studies were performed using murine anti-human E-cadherin (Clone 67A4; Immunotech), murine anti-human ICAM-1 (Clone84H10; Serotec), murine anti-human CD45 : RPE (Clone T29/33; DAKO), murine anti-human CD103 (Clone Ber-ACT8; DAKO), murine anti-human CD3 (OKT3; Janssen–Cilag, Buckinghamshire, UK), murine anti-human CD11a (α subunit of LFA-1, Clone MHM24; DAKO), murine anti-human CD18 (β subunit of LFA-1, Clone MHM23; DAKO), murine IgG_1_ (isotype control X0931, DAKO).

### Cell culture

A pancreatic carcinoma cell line, Panc-1, was used in these experiments. Panc-1 was developed from an undifferentiated pancreatic carcinoma of ductal origin (Lieber *et al*, 1975) (ECACC No 87092802; ECACC, Porton Down, UK). Cell lines were cultured in DMEM complete medium (Life Technologies, Paisley, UK) containing 10% foetal calf serum (Sigma, UK), penicillin and streptomycin (Life Technologies). Cell lines were routinely passaged when confluent. Transfected Panc-1 cells were cultured as above with the addition of hygromycin B (350 μg ml^−1^). Medium (including hygromycin B) was replaced every 2 days. Epstein Barr virus transformed B-lymphocytes (EBV-BCL) were obtained from ECACC. These were cultured in RPMI 1640 complete medium (Life Technologies) containing 10% foetal calf serum (Sigma, UK), penicillin and streptomycin (Life Technologies).

### Preparation of CD103+ lymphocytes

Fresh peripheral blood lymphocytes (PBL) were obtained from normal healthy volunteers. Whole blood was diluted with an equal volume of RPMI 1640. The resultant suspension was divided into 10 ml aliquots and separated by density centrifugation over Ficoll-Paque (Nycomed, UK) at 400 **g** for 25 min. The interfacial layer consisting of mononuclear cells was recovered and washed in RPMI 1640. The pellet of cells was re-suspended in complete medium and incubated in a horizontal 25 cm^−2^ tissue culture flask (Corning, UK) for 1 h to allow the monocyte population to adhere to the plastic. The non-adherent lymphocyte population was gently re-suspended, removed and the cells counted. From this population, T-lymphocytes were selected using an immunomagnetic T cell negative isolation kit (Dynal, Wirral, UK) in order to remove natural killer cell activity. Briefly, cells were incubated with an antibody mixture containing antibodies specific for CD14 (Macrophages), CD16 (Granulocytes and NK cells), CD56 (NK cells), HLA class II DR/DP (B cells). Subsequent incubation with Dynabeads captured the antibody-bound cells. The coated cells were then separated with a magnet and discarded, leaving a pure T lymphocyte population.

Irradiated EBV-BCL (50 Gy; ^137^Cs source) were added to the T lymphocyte suspension at a ratio of 2 : 1 (stimulators : responders). This one-way MLR was divided into two 25 cm^−2^ tissue culture flasks, which were incubated at 45° to the horizontal at 37°C for 5 days. This step allows activation and proliferation of allo-specific PBL to provide large numbers of activated human T lymphocytes. After 5 days recombinant TGFβ_1_ (R&D Systems, UK) was added to one flask at a concentration of 3 ng ml^−1^. Incubation of both flasks continued at 37°C until day 8 or 9 when the cells were retrieved. The viable population was separated from debris by further density centrifugation before being utilised for phenotypic and functional analysis.

### Transfection

Panc-1 cells were transfected using a modified pCEP4 plasmid vector containing human DNA coding for E-cadherin (a kind gift from JMG Higgins, Boston, MA, USA). The plasmid was transfected into Panc-1 cells using SuperFect (Qiagen, Crawley, UK). Two micrograms of plasmid DNA and 8 μl of liposomes dissolved in DMEM (serum and antibiotic free) was used for each transfection according to the Qiagen protocol. Briefly the DNA/liposome mixture was allowed to stand for 15 min at room temperature. Semi-confluent Panc-1 cells in a six well plate were washed with 0.01 M Phosphate buffered saline (PBS) and 600 μl of complete DMEM was added to the DNA/liposome mixture above which was then added to each well. After incubation at 37°C for 3 h the cells were gently washed with PBS and replaced with complete DMEM. After 24 h the transfected cells were selected using 350 μg ml^−1^ hygromycin B in complete DMEM. A study to determine the selection conditions for the parental cell line showed that 350 μg ml^−1^ of hygromycin B killed all wild-type cells after 7 days (data not shown). Transfected colonies were isolated and sub-cultured to allow phenotypic analysis.

### Phenotypic analysis

Panc-1 cells (transfected and wild-type) were analysed for expression of the cell adhesion molecules E-cadherin and ICAM-1 and also to confirm that CD45 (a pan T cell marker) was not expressed. Cells were grown to confluence in 75 cm^−2^ tissue culture flasks (Corning) and then supernatant and non-viable cells removed. Cells were detached from plastic by brief incubation with a 3 mM solution of EDTA in PBS because Trypsin caused cleavage of E-cadherin and resulted in inconsistent detection of E-cadherin expression on cell lines. After detachment the cells were washed and counted. 5×10^5^ cells were added to each FACS tube, washed again and re-suspended in 50 μl of 5% foetal calf serum (FCS) in PBS. Primary antibody was added and incubated at 4°C for 30 min. The cells were washed again and 50 μl of FITC conjugated goat anti-mouse secondary antibody added. This step was omitted for conjugated primary antibodies. A further 30 min incubation at 4°C was followed by washing and re-suspension of cells in 5% FCS in PBS. The cells were then analysed by two-colour flow cytometry. For flow cytometric analysis, the viable population was routinely identified by propidium iodide (PI) exclusion; 5 μl of a 25 μg ml^−1^ stock solution of PI in distilled water was added to 5×10^5^ cells in 200 μl PBS immediately prior to analysis.

The efficiency of Dynabead T lymphocyte negative selection was assessed by assaying the selected population for CD3 expression. T-cells in the MLR (Day 8/9) were assayed similarly for expression of CD45, CD103, CD11a (α chain LFA-1) and CD18 (β chain LFA-1).

### Flow cytometric adhesion assay

A sensitive and reproducible flow cytometric adhesion assay previously developed by our group was modified for this study (Korlipara *et al*, 1996). Briefly, 2×10^5^ pancreatic cancer cells (transfected or wild-type) were seeded onto a 24-well tissue culture grade plate (Corning) and allowed to grow to confluence over 24 h. The plate was washed and 5×10^5^ MLR-derived lymphocytes were added to each well of the 24-well plate in RPMI 1640. For assay of specific adhesion blockade, the lymphocytes were incubated for 30 min at 37°C with an appropriate blocking antibody at a pre-optimised concentration before addition to the adhesion assay plates; for control an isotype-matched antibody was used at the same concentration.

In all cases the lymphocytes were allowed to contact the cancer cells for 1 h at 37°C. After this time the non-adherent cells were re-suspended by use of a mechanical plate shaker for 5 min at 150 oscillations per minute (IKA-Shuttler MTS4; NE Lab supplies, UK). The plate was then gently washed three times with PBS to remove any non-adherent cells and 3 mM EDTA in PBS used to detach cells from each other and the plate to produce a single cell suspension. The cell suspension was added to FACS tubes, centrifuged and re-suspended in 50 μl of 5% FCS in PBS. A total of 2.5 μl of PE-conjugated anti-CD45 antibody was added to each tube (to label the lymphocytes) and incubated for 30 min at 4°C. The cells were then washed again and analysed by flow cytometry. The cell populations were distinguished by size (forward scatter) and fluorescence channel 2 (CD45-PE) intensity. Ten replicates of each experiment were performed and a mean and s.e.m. of the ratio of lymphocytes to pancreatic cells calculated. Student's unpaired *t*-test was used to compare adhesion between different populations.

### Chromium release cytotoxicity assay

The lytic activity of CD103+ lymphocytes was assessed in an 8 h ^51^Cr-release assay. Panc-1 cells were detached with 3 mM EDTA and a pellet of carcinoma cells was resuspended at 37°C with 100 μCi [^51^Cr] sodium chromate (CJS-4; Amersham International, UK) per 10^6^ cells for 90 min with agitation every 15 min. The cells were re-suspended in 20 ml RPMI 1640 and incubated for 30 min at 37°C before being washed five times by centrifugation. Five thousand tumour cells were incubated with MLR lymphocytes at effecter : target ratios ranging from 100 : 1 to 25 : 1 in round-bottomed 96-well microtiter plates. After 8 h, 100 μl of supernatant of each well was collected, and radioactivity was measured with a gamma counter (Wallac γ counter). Specific lysis was calculated using the formula: specific ^51^Cr-release=[(experimental counts – spontaneous counts)/(maximal counts – spontaneous counts)×100%]. Some T cells were re-activated prior to use in the cytotoxicity assay, by incubation at 37°C for 1 h with anti-CD3 antibody. To determine CD103 restriction of tumour cell lysis, in some assays the target cells were pre-incubated with a CD103 blocking antibody or murine IgG_1_ isotype control at optimal concentration pre-determined using the adhesion assay.

## RESULTS

### Phenotypic analysis

#### Pancreatic cancer cell lines

Figure 1

**Figure 1:**
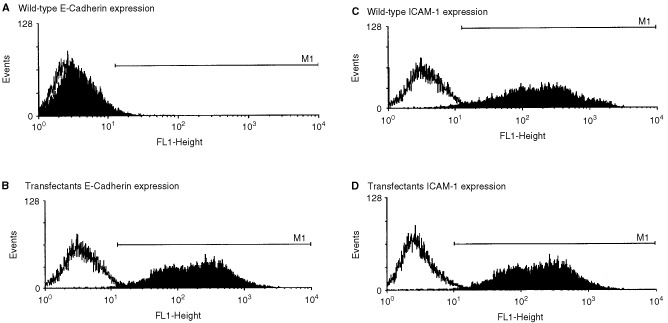
Flow cytometric analysis of the expression of adhesion molecules by Panc-1 cells (wild-type and E-cadherin transfected cells). The filled histograms show experimental staining; the unfilled histograms show results from isotype matched control antibody staining. The marker (M1) includes 3% of the control stained cells and defines positive antigen expression.

shows the results of flow cytometric analysis of adhesion molecule expression by viable Panc-1 cells. Figure 1a shows that the wild-type Panc-1 cells expressed no cell-surface E-cadherin. However, the stably transfected Panc-1 cells homogeneously expressed high levels of this adhesion molecule. Figure 1c,d show that both wild-type and E-cadherin transfected Panc-1 cells expressed equally high levels of cell-surface ICAM-1. In all cases a 3 mM solution of EDTA in PBS was used to detach the cells from culture plastic as treatment with trypsin was found to cleave E-cadherin from the cells resulting in inconsistent detection of E-cadherin expression. Neither wild-type nor the transfected cells expressed CD45 (data not shown).

#### Lymphocytes

Lymphocyte phenotype was analysed by flow cytometry. Each analysis was performed on three separate occasions. Non-viable lymphocytes always comprised fewer than 5% of the population and were excluded from analysis on the basis of propidium iodide (PI) staining. Routine analysis of the results of immunomagnetic T cell selection showed that CD3+ve cells made up more than 99% of the negatively selected population.

Figure 2a

**Figure 2:**
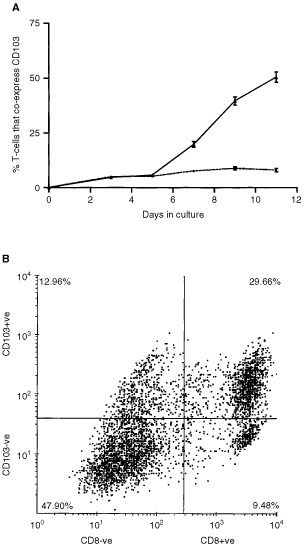
Flow cytometric examination of the induction of CD103 expression by T cells in MLR culture. (**A**) Shows the time course for induction of CD103 on lymphocytes cultured in MLR with TGFβ_1_ added on day 3 (solid line) or without TGFβ_1_ (dotted line). The data points show the mean of 10-fold determinations; the error bars show the s.e.m. (**B**) Shows results from a representative two-parameter flow cytometric dot-plot for viable cells harvested from a day 9 MLR which was supplemented with TGFβ_1_ on day 3. It can be seen that the majority (75.6%) of the CD8 T cells at this time point also express CD103.

shows a representative time-course for the induction of CD103 expression following addition of TGFβ_1_ to an MLR culture at day 3. Serial harvest and phenotypic analyses of the T cells showed that at day 9 the CD103+ population had increased to a mean of 39.7%, and by day 11 to a mean of 50.6%. Lymphocytes from MLR without addition of TGFβ_1_ showed only a slight increase in CD103 expression, which reached a plateau of 8.8% by day 9 of culture. Figure 2b shows the results of a further phenotypic characterisation performed 9 days after the initiation of MLR culture in the presence of TGFβ_1_. The 2-parameter flow cytometric dot-plot shows that 42.6% of the T cells expressed CD103, that 39.1% of the T cells were CD8+ve and that 75.9% of the CD8+ve T cells also expressed CD103. Fewer than 22% of the CD8-ve T cells expressed CD103, suggesting that CD103 is preferentially expressed by class I MHC-restricted cytotoxic T cells. Additional experiments (data not shown) demonstrated that TGFβ_1_ did not alter the proportion of CD8+ T cells in the MLR (37% in representative non-TGFβ_1_-supplemented cultures) but that fewer than 5% of these cells expressed CD103. Furthermore, the T cells in both TGFβ_1_ supplemented and non-supplemented cultures expressed similarly high levels of both the CD11a and CD18 integrin-chains of LFA-1. The leukocyte common antigen CD45 was also expressed by all T cells in both cultures.

### Flow cytometric adhesion assays

Figure 3

**Figure 3:**
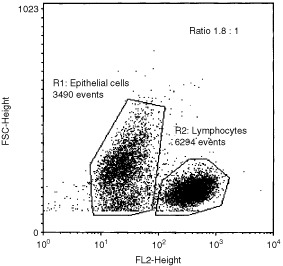
Representative two-parameter dot-plot from a typical flow cytometric analysis of the mixed T cell and epithelial cell population derived from one well of a 24-well adhesion assay plate. The analysis regions allow clear discrimination between the epithelial cells (R1) and lymphocytes (R2) and calculation of a lymphocyte : epithelial cell binding ratio of 1.8.

shows the results from a typical flow cytometric analysis of the mixed T cell and epithelial cell population derived from one well of a 24-well adhesion assay plate. The two-parameter dot-plot shows a clear discrimination between the CD45+ve T cells (delineated by R2) and the population of larger, but CD45-ve epithelial cells (delineated by R1). Following enumeration of the cells in each region, the lymphocyte: epithelial cell binding ratio (R2/R1) can be calculated.

Figure 4a

**Figure 4:**
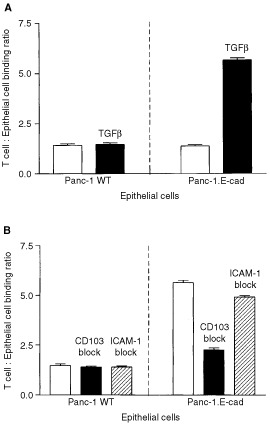
Examination of the adhesion of activated T lymphocytes to Panc-1 cells. (**A**) Demonstration of binding of T cells from non-TGFβ_1_ supplemented (open bars) and TGFβ_1_-supplemented (solid bars) MLR cultures to wild-type and E-cadherin transfected Panc-1 cells. The bars show the mean of 10-fold determinations; the error bars show the s.e.m. (**B**) Examination of antibody-blockade of the adhesion of T cells from a TGFβ_1_ supplemented MLR to wild-type and E-cadherin transfected Panc-1 cells. Control assays are indicated by open bars, solid bars show blockade of CD103 and hatched bars show blockade of ICAM-1. The bars show the mean of 10-fold determinations; the error bars show the s.e.m.

shows that addition of TGFβ_1_ during MLR culture, and hence up-regulation of CD103 expression, made no difference to the binding of day 9 T cells to wild-type Panc-1 cells (*P*>0.6). However, T cells from TGFβ_1_ supplemented MLR cultures showed a significantly increased binding to E-cadherin expressing Panc-1 transfectants (*P*<0.0001).

The binding of T cells from TGFβ_1_ supplemented day 9 MLR to wild-type Panc-1 cells was not inhibited by antibody-mediated blockade of either CD103 or ICAM-1 (*P*>0.2; Figure 4b). However, antibody-blockade of CD103 reduced the adhesion of these cells to E-cadherin expressing Panc-1 transfectants to near basal levels (*P*<0.001). Significantly, blockade of ICAM-1 had no effect on T cell adhesion to the transfected Panc-1 cells (*P*>0.2; Figure 4b).

### Chromium release cytotoxicity assay

Figure 5a

**Figure 5:**
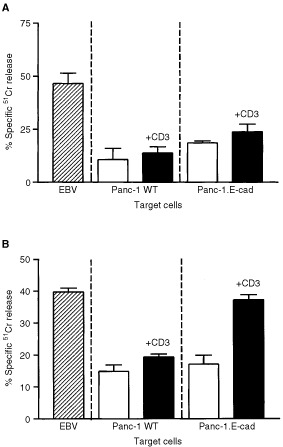
Examination of cytolysis of Panc-1 cells by activated T cells with or without additional T cell receptor stimulation by CD3. (**A**) Assay performed using T cells from a day 9 MLR in the absence of exogenous TGFβ_1_. Hatched bar shows control cytolysis of the antigen-presenting cells used for T cell activation, open bars show Panc-1 lysis in the absence of CD3 stimulation and solid bars show the lysis produced following CD3 stimulation. The bars show the mean of 10-fold determinations; the error bars show the s.e.m. (**B**) Results of a similar assay performed using T cells harvested from an MLR performed in the presence of exogenous TGFβ_1_. The bars show the mean of 10-fold determinations; the error bars show the s.e.m.

shows representative results from a ^51^Cr release cytotoxicity assay using T cells harvested from a non-TGFβ_1_ supplemented day 9 MLR. The assay was routinely performed across a range of effecter: target cell ratios, but 50 : 1 regularly produced optimal cytotoxicity and these data are presented. The allo-specific T cells produced efficient positive control lysis of the EBV cell line used to stimulate the MLR. However, little background lysis of either wild-type or E-cadherin expressing Panc-1 transfectants was observed. T cell re-activation with anti-CD3 increased only slightly the lysis of these 2 cell lines (Panc-1 WT, *P*>0.09; Panc-1.E-cad *P*>0.8; Figure 5a).

The results of a similar experiment performed using T cells from a TGFβ_1_ supplemented MLR are shown in Figure 5b. In this experiment, stimulation of CD3 failed to increase the limited (<20%) lysis of the wild-type targets (*P*>0.15), but greatly enhanced cytolysis of the E-cadherin expressing Panc-1 transfectants to a level comparable with that of the specific EBV target cells (>35%; *P*<0.0001).

**Figure 6:**
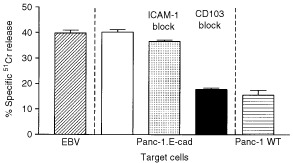
Investigation of the effect on Panc-1 cytolysis of antibody blockade of ICAM-1 or CD103. The effector T cells were harvested from an MLR performed in the presence of TGFβ_1_ and were additionally stimulated with anti-CD3 antibody prior to addition to the target cells. The hatched bar shows control cytolysis of the antigen-presenting cells used for T cell activation, the open bar shows the lysis of E-cadherin transfected Panc-1 in the presence of a control antibody, the shaded bar shows the effect of ICAM-1 blockade, the solid bar shown the effect of CD103 blockade and the horizontally filled bar shows lysis of wild-type Panc-1 cells. The bars show the mean of 10-fold determinations; the error bars show the s.e.m.

To verify the function of adhesion between CD103 and E-cadherin in the lysis of pancreatic cancer cells, a further series of cytolysis experiments was performed in the presence of CD103 antibody blockade. Figure 6

shows results from a representative assay in which blockade of CD103 reduced the lysis of E-cadherin expressing Panc-1 cells by T cells from a TGFβ_1_ supplemented MLR (*P*<0.0001) to levels comparable with the lysis of wild-type target cells (*P*>0.08). ICAM-1 blockade did not reduce the lysis of E-cadherin expressing Panc-1 cells (p>0.1). Control lysis of the specific EBV transformed target cells was not inhibited by CD103 blockade.

## DISCUSSION

Immunohistological studies of the distribution of T cells in normal and inflamed pancreatic tissue suggest that CD103+ lymphocytes play an important role in the pancreatic epithelium (Ademmer *et al*, 1998; Ebert *et al*, 1998). Staining of serial sections showed that infiltrating T cells included both CD4+ (helper) and CD8+ (cytotoxic) phenotypes and were distributed in the interstitial tissue, periductal regions, or intercalating between the epithelial cells. Large numbers of lymphocytes were seen in inflamed and malignant pancreas, where CD4+CD103- T cells form the predominant subset. However, CD103+ lymphocytes, generally co-expressing CD8, were observed in the intraepithelial compartment, implying that this subset is analogous to gut IEL (Ademmer *et al*, 1998; Ebert *et al*, 1998).

Induction of CD103 on lymphocytes from peripheral blood was achieved in this study by activation of allospecific T cells in an MLR, followed by culture in the presence of TGFβ_1_; the MLR system was chosen to model antigen-specific activation of large numbers of primary human T cells. The results from this model are consistent with previous studies (Austrup *et al*, 1995), although the methodology described in the current report is novel. Previous *in vitro* studies demonstrate that the majority of T cells of the CD103 phenotype are activated CD8+ cells (Cerf-Bensussan *et al*, 1987; Rihs *et al*, 1996; Ademmer *et al*, 1998; Fujihara *et al*, 1999). The population produced *in vitro* using the technique in the current study was essentially similar to IEL with regards to activation and predominance of the CD8 phenotype. Therefore, in principle, these cells can be used to model IEL in terms of phenotype and adhesive properties.

The important components of the system used to generate CD103+ T cell *in vitro* were activation of the lymphocytes and, crucially, the addition of TGFβ_1_. The T cells found in the pancreas are predominantly CD45RO positive (Ebert *et al*, 1998), indicating prior activation. It has also been shown previously that the CD103 positive IEL isolated from gut epithelium express surface markers consistent with prior activation (Schieferdecker *et al*, 1990). It is likely that activated IEL acquire the CD103 phenotype *in vivo* in response to the presence of high levels of TGFβ_1_ in the epithelial microenvironment. This is consistent with the high levels of TGFβ_1_ observed in pancreatic adenocarcinoma (Friess *et al*, 1993) and chronic pancreatitis (van Laethem *et al*, 1995).

Activation of T cells followed by addition of TGFβ_1_ significantly increased adhesion to an E-cadherin transfected pancreatic carcinoma cell line; this treatment had no effect on adhesion to E-cadherin deficient wild-type Panc-1 cells. This observation suggests that the CD103→E-cadherin interaction is important for lymphocyte adhesion to Panc-1 target cells. However, it is possible that target cell transfection and treatment with TGFβ_1_ altered T cell adhesion properties by some alternative mechanism. In order to address this a series of experiments was performed to define the effect of blockade of the CD103 integrin with an excess of anti-CD103 antibody. This treatment abrogated the enhanced adhesion to E-cadherin expressing targets, confirming that the increased adhesion to E-cadherin expressing targets was due to the expression of functional CD103.

Normal pancreatic epithelium expresses high levels of E-cadherin, which is associated with the plasma membrane (Weinel *et al*, 1996). These cells do not express ICAM-1 (Shimoyama *et al*, 1997) but this molecule can be induced by treatment with the proinflammatory cytokines TNFα and IFNγ (Schwaeble *et al*, 1993). The LFA-1→ICAM-1 interaction is known to be important for adhesion of lymphocytes to vascular endothelium as a precursor to extravasation and penetration of the extra-vascular space (Springer, 1990). Hence, expression of ICAM-1 within the pancreatic epithelium could allow T cell adhesion in the absence of E-cadherin.

Both CD103+ and CD103- T cells showed a small but similar adhesion to E-cadherin negative wild-type Panc-1 cells. Phenotypic analysis of the Panc-1 cells showed high levels of ICAM-1 expression, whilst both CD103+ and CD103- T cells expressed high levels of LFA-1. These findings suggest that the LFA-1→ICAM-1 interaction could stabilise T cell adhesion to Panc-1 cells. However, adhesion studies performed in the presence of anti-ICAM-1 blockade did not significantly reduce either CD103+ or CD103- T cell adhesion to E-cadherin negative wild-type Panc-1 cells, suggesting that LFA-1 does not enhance adhesion in this system.

T-cell-mediated target cell lysis can be modelled in the absence of a specific antigen by re-stimulating activated T cells with CD3 antibodies immediately prior to ^51^Cr-release assay (Roberts and Kilshaw, 1993). This ‘re-directed lysis’ technique was used in the current study to assess specific cytolysis of Panc-1 cells using MLR-activated T cells as an effecter cell population. It was found that E-cadherin expressing Panc-1 transfectant cells were efficiently lysed following re-stimulation of CD103+ T cells produced by TGFβ_1_ treatment; these effecter lymphocytes did not lyse wild-type Panc-1 cells. Furthermore, it was found that restimulation with anti-CD3 antibodies was essential for target cell lysis, indicating a requirement for specific T cell receptor stimulation. These observations suggest that the CD103→E-cadherin interaction is essential for specific T cell-mediated lysis of pancreatic cancer cell targets. However, it is possible that transfection of the Panc-1 cells altered their susceptibility to lysis by an alternative mechanism. To test this possibility, assays were performed in which the CD103 integrin was blocked by addition of anti-CD103 antibodies. This treatment abrogated the enhanced lysis of E-cadherin expressing targets, confirming that the increased lysis of E-cadherin expressing Panc-1 target cells was produced by specific adhesion of CD103+ T cells.

Immunohistochemical studies have demonstrated that pancreatic carcinoma *in vivo* has reduced E-cadherin expression when compared to normal pancreas, and that loss of this antigen correlates with advanced stage, high grade and the presence of lymph node metastasis (Pignatelli *et al*, 1994). In the current study it has been demonstrated that re-expression of E-cadherin by an E-cadherin negative pancreatic carcinoma cells is necessary both for the adhesion of CD103-expressing lymphocytes and the susceptibility of these cancer cells to subsequent lysis. Hence, the decreased expression of E-cadherin by pancreatic carcinoma cells *in vivo* could reduce or abolish the ability of tumour antigen-specific T-lymphocytes in the TGFβ-rich cancer microenvironment to adhere to and lyse their targets, allowing cancer cells to escape from normal intraepithelial immunological surveillance. This suggests a mechanism for immunological selection of cancer cells with a reduced E-cadherin expression (Seymour *et al*, 1999; Pettit *et al*, 2000). The results of our study have implications for the design of immunotherapeutic strategies based on T cell killing of pancreatic cancer cells within a TGFβ-rich microenvironment, as this process may be highly dependent on T cell adhesion to E-cadherin.
